# Association between computed tomography obstruction index and mortality in elderly patients with acute pulmonary embolism: A prospective validation study

**DOI:** 10.1371/journal.pone.0179224

**Published:** 2017-06-08

**Authors:** Marie Méan, Tobias Tritschler, Andreas Limacher, Stéphane Breault, Nicolas Rodondi, Drahomir Aujesky, Salah D. Qanadli

**Affiliations:** 1 Department of General Internal Medicine, Inselspital, Bern University Hospital, University of Bern, Bern, Switzerland; 2 Division of General Internal Medicine, Lausanne University Hospital, Lausanne, Switzerland; 3 CTU Bern, and Institute of Social and Preventive Medicine (ISPM), University of Bern, Bern, Switzerland; 4 Department of Radiology, Lausanne University Hospital, Lausanne, Switzerland; 5 Institute of Primary Health Care (BIHAM), University of Bern, Bern, Switzerland; National and Kapodistrian University of Athens, GREECE

## Abstract

**Introduction:**

Computed tomography pulmonary angiography (CTPA) has not only become the method of choice for diagnosing acute pulmonary embolism (PE), it also allows for risk stratification of patients with PE. To date, no study has specifically examined the predictive value of CTPA findings to assess short-term prognosis in elderly patients with acute PE who are particularly vulnerable to adverse outcomes.

**Methods:**

We studied 291 patients aged ≥65 years with acute symptomatic PE in a prospective multicenter cohort. Outcomes were 90-day overall and PE-related mortality, recurrent venous thromboembolism (VTE), and length of hospital stay (LOS). We examined associations of the computed tomography obstruction index (CTOI) and the right ventricular (RV) to left ventricular (LV) diameter ratio with mortality and VTE recurrence using survival analysis, adjusting for provoked VTE, Pulmonary Embolism Severity Index (PESI), and anticoagulation as a time-varying covariate.

**Results:**

Overall, 15 patients died within 90 days. There was no association between the CTOI and 90-day overall mortality (adjusted hazard ratio per 10% CTOI increase 0.92; 95% confidence interval [CI] 0.70–1.21; *P* = 0.54), but between the CTOI and PE-related 90-day mortality (adjusted sub-hazard ratio per 10% CTOI increase 1.36; 95% CI 1.03–1.81; *P* = 0.03). The RV/LV diameter ratio was neither associated with overall nor PE-related 90-day mortality. The CTOI and the RV/LV diameter ratio were significantly associated with VTE recurrence and LOS.

**Conclusions:**

In elderly patients with acute PE, the CTOI was associated with PE-related 90-day mortality but not with overall 90-day mortality. The RV/LV diameter ratio did not predict mortality. Both measures predicted VTE recurrence and LOS. The evaluated CTPA findings do not appear to offer any advantage over the PESI in terms of mortality prediction.

## Introduction

The prognosis of acute pulmonary embolism (PE) is highly variable [1, 2], and various clinical and radiological parameters have been evaluated to help physicians to risk stratify patients with acute PE [3–5]. Given that computed tomography pulmonary angiography (CTPA) has become the method of choice for diagnosing acute PE, risk stratification based on initial imaging would combine diagnosis and prognostic assessment into a single test. To assess short-term prognosis in patients with PE, several CTPA findings have been evaluated for their predictive value [6–9]. The best-validated scoring system taking into account the embolic burden assessed by CTPA is the computed tomography obstruction index (CTOI) [10]. The CTOI is calculated by adding the number of occluded segmental arteries after assigning a weighting factor depending on the degree of obstruction to each occluded artery [10]. Several studies evaluated the score for its prognostic ability with controversial results [11–16]. Most validation studies were limited by retrospective or single-center design [11–13], or small sample size [14, 15].

Besides the CTOI, the most commonly used CTPA parameter for assessing short-term prognosis is the right ventricular (RV) to left ventricular (LV) diameter ratio [11, 13, 17, 18]. However, low likelihood ratios (LR) limit its ability to risk classify patients with PE (positive LR 1.27, negative LR 0.71) [8].

Even though advanced age is associated with an increase in the incidence and overall mortality of PE [19–22], to our knowledge, no study has specifically examined the association between CTPA findings and mortality in elderly patients. Given that co-morbid conditions are more frequent in elderly patients and cardiac dimensions alter with increasing age even without underlying diseases [23], risk stratification by CTPA findings may differ for elderly patients. We therefore aimed to (1) prospectively evaluate the prognostic performance of the CTOI and the RV/LV diameter ratio in a multicenter cohort of elderly patients with acute PE and (2) to compare the prognostic accuracy of the CTOI, the RV/LV diameter ratio, and the Pulmonary Embolism Severity Index (PESI), a validated clinical prognostic score for acute PE [4].

## Methods

### Study design and patients

The study was conducted between September 2009 and December 2013 as part of the SWIss venous Thromboembolism COhort (SWITCO65+), a prospective, multicenter cohort study to assess long-term medical outcomes in elderly patients with acute symptomatic venous thromboembolism (VTE) from all five university and four high-volume non-university hospitals in Switzerland [24]. Consecutive patients aged 65 years or older with objectively diagnosed, symptomatic VTE were identified in the in- and outpatient services of all participating study sites. For this study, we only considered patients with objectively diagnosed, acute symptomatic PE, defined as positive CTPA in patients with acute chest pain, new or worsening dyspnea, hemoptysis, or syncope [25]. Patients with PE who did not undergo CTPA were excluded from the present analysis.

Exclusion criteria were inability to provide informed consent (e.g., severe dementia), conditions incompatible with follow-up (e.g., terminal illness, geographic inaccessibility), thrombosis at a different site than lower limb, catheter-related thrombosis, or previous enrolment in the cohort.

Treatment of VTE, including type and duration of anticoagulant therapy, invasive treatments (i.e. thrombolysis, insertion of a vena cava filter), and clinical follow-up was entirely left to the discretion of the managing physicians.

Informed consent was obtained from all participants. The ethics committee at each participating center approved the study. The approving ethics committees were the "Commission cantonale d'éthique de la recherche sur l'être humain Vaud" (site of Lausanne), "Commission cantonale d'éthique de la recherche Genève" (site of Geneva), "Kantonale Ethikkommission Bern" (site of Bern), "Kantonale Ethikkommission Zürich" (site of Zurich), "Ethikkommission Nordwest- und Zentralschweiz" (sites of Basel, Lucerne and Baden), "Ethikkommission des Kantons Thurgau" (site of Frauenfeld) and "Ethikkommission des Kantons St. Gallen" (site of St. Gallen). A detailed description of the study methods has been published previously [24].

### Baseline data collection and follow-up

Trained study nurses prospectively collected baseline demographics (age and gender), co-morbid conditions (active cancer, arterial hypertension, diabetes mellitus, acute or chronic heart failure, chronic pulmonary disease, cerebrovascular disease, chronic liver disease, chronic renal failure), history of VTE, type of PE (provoked versus cancer-related versus unprovoked), vital signs (mental status, heart rate, blood pressure, temperature, respiratory rate, and arterial oxygen saturation), routine laboratory findings (hemoglobin and serum creatinine), concomitant antiplatelet therapy and VTE-related treatment using standardized data collection forms.

Follow-up included one telephone interview and two surveillance face-to-face evaluations during the first year of study participation and then semi-annual contacts, alternating between face-to-face evaluations (clinic visits or home visits in house-bound patients) and telephone calls as well as periodic reviews of the patient’s hospital chart.

### CT obstruction index and RV/LV diameter ratio

CTPA was performed in each participating study center, recorded on compact discs, and anonymously sent to Lausanne University Hospital where two certified radiologists evaluated them independently. Disagreement was resolved by consensus. The radiologists were blinded to patients’ baseline characteristics and treatments.

To calculate the CTOI, the arterial tree of each lung was considered to have 10 segmental arteries (three in the upper lobes, two in the middle lobe and in the lingula, and five in the lower lobes). The presence of an embolus in a segmental artery was scored 1 point. Central or paracentral emboli were scored a value equal to the number of segmental arteries arising distally. Depending on the degree of vascular obstruction a weighting factor was assigned to each value (0, no thrombus; 1, partial occlusion; and 2, total occlusion). Isolated subsegmental embolus was considered as a partially occluded segmental artery and was assigned a value of 1. Thus, the CTOI could vary from 1 to 40 points per patient. Dividing the patient score by the maximal total score and multiplying the result by 100 calculated the percentage of vascular obstruction. Based on the percentage of vascular obstruction, patients were then divided into three groups (<15% versus 15–50% versus >50%).

In a second step, RV dysfunction was assessed by measuring the ratio between the RV diameter and the LV in 4-chamber view (RV/LV). A RV/LV diameter ratio of >0.9 was defined as right ventricular dysfunction [26].

### Assessment of the PESI

The PESI is a validated prognostic score for patients with acute PE and comprises 11 easily available clinical variables, including patient demographics, comorbid diseases, and vital signs [4]. Based on patient demographics and the first available baseline clinical data obtained by chart review, we determined the presence of the prognostic variables comprising the PESI. Whenever necessary, missing values were assumed to be normal. This strategy is widely used in the clinical application of prognostic models and reflects the methods used in the original derivation of the PESI [4, 27].

### Study outcome

The primary outcome was overall mortality within 90 days of PE diagnosis. We assessed the clinical outcomes using patient or proxy interviews, interview of the patient’s primary care physician, and/or hospital chart review. A committee of three blinded, independent clinical experts adjudicated the cause of death. Death was judged to be a definite fatal PE if it was confirmed by autopsy, or if death followed a clinically severe PE, either initially or after an objectively confirmed recurrent event. Death in a patient who died suddenly or unexpectedly was classified as possible fatal PE. Final classification was made on the basis of the full consensus of this committee.

Secondary outcomes were PE-related mortality within 90 days, the recurrence of an objectively confirmed, symptomatic VTE during the whole follow-up, defined as a fatal or new non-fatal PE or new deep vein thrombosis [24], and the length of hospital stay (LOS) of patients who were hospitalized for the index PE.

### Statistical analyses

We compared baseline and procedural characteristics of patients by level of the CTOI using the chi-squared test and the non-parametric Kruskal-Wallis rank test as appropriate. We compared the cumulative overall mortality, PE-related mortality, and recurrence of VTE among patients with different levels of the CTOI using Kaplan-Meier curves and the log-rank test.

We examined associations of the CTOI, the RV/LV diameter ratio, and the PESI with the time to death using Cox-regression with robust standard errors. For VTE recurrence and PE-related death, we used competing risk regression according to Fine and Gray [28], accounting for non-PE-related death as a competing event. The strength of the association is reflected by the sub-hazard ratio (SHR), which is the ratio of hazards associated with the cumulative incidence function in the presence of a competing risk.

Due to low event numbers, only a minimal adjustment could be performed. Associations of the CTOI and the RV/LV diameter ratio with clinical outcomes were adjusted for provoked PE, the PESI, and anticoagulation treatment as a time-varying covariate. Associations of the PESI with clinical outcomes were adjusted for provoked VTE and anticoagulation treatment as a time-varying covariate.

We compared the discriminative power of the CTOI, the RV/LV diameter ratio, and the PESI to predict mortality and VTE recurrence using Harrell’s C concordance statistic. We assessed the association of the CTOI, the RV/LV diameter ratio, and the PESI with LOS among patients admitted due to the index PE event using a shared-frailty lognormal survival model accounting for variation of LOS between study sites. LOS was censored if a patient died in hospital. Associations were adjusted for age, gender, type of PE (unprovoked versus provoked versus cancer-related), body mass index (BMI), prior VTE, central PE, concomitant deep vein thrombosis (DVT), arterial hypertension, diabetes mellitus, heart failure, chronic pulmonary disease, cerebrovascular disease, chronic liver disease, chronic renal failure, the PESI, and antiplatelet therapy/non-steroidal anti-inflammatory drugs.

We considered *P*-values <0.05 to be statistically significant. All analyses were done using Stata 14 (Stata Corporation, College Station, Texas).

## Results

Of the 316 consenting patients with available CTPA, we excluded 24 patients with inadequate CTPA quality and one patient with cancer-related non-thrombotic obstruction, leaving a final study sample of 291 patients with acute PE. Analyzed patients had a median age of 75 years (interquartile range [IQR] 69–81), 138 (47%) were women, 69 (24%) had provoked and 45 (15%) had cancer-related VTE (Table 1). Overall, 29%, 55%, and 16% of patients had a CTOI of <15%, 15–50%, and >50%, respectively. Patients with a higher CTOI were significantly younger (*P* = 0.01), had more often an unprovoked PE (*P* = 0.047), a higher BMI (*P* = 0.01), a higher rate of thrombolysis (*P* <0.001), a longer duration of anticoagulation (*P* = 0.003) and a higher RV/LV diameter ratio (*P* <0.001) (Table 1). Median follow-up was 31 months (IQR 24–42 months). Overall, 5% (15/291) of patients died within 90 days (6 from definite or possible PE). During the whole follow-up, 12% (34/291) of patients had a recurrent VTE (16 patients within 12 months). Median LOS was 8 days (IQR 5–12 days).

**Table 1:** Patient baseline characteristics by CTOI.

	All (N = 291)	CTOI <15% (N = 84)	CTOI 15–50% (N = 159)	CTOI >50% (N = 48)	*P*-value
	n (%) or median (interquartile range)				
**Age (years)^a^**	75.0 (69.0; 81.0)	77.0 (70.0; 82.0)	74.0 (70.0; 83.0)	72.5 (67.0; 76.5)	0.01
**Female sex**	138 (47)	45 (54)	67 (42)	26 (54)	0.14
**Type of PE**					0.03
**Unprovoked^b^**	177 (61)	42 (50)	102 (64)	33 (69)	
**Provoked^c^**	69 (24)	28 (33)	29 (18)	12 (25)	
**Cancer-related^d^**	45 (15)	14 (17)	28 (18)	3 (6)	
**BMI (kg/m^2^)^a^**	26.7 (24.2; 30.1)	26.2 (24.2; 29.4)	26.4 (23.9; 29.7)	29.1 (25.2; 33.7)	0.01
**Prior VTE**	81 (28)	17 (20)	46 (29)	18 (38)	0.09
**Arterial hypertension**	192 (66)	53 (63)	104 (65)	35 (73)	0.51
**Diabetes mellitus**	39 (13)	12 (14)	17 (11)	10 (21)	0.19
**Acute or chronic heart failure^e^**	39 (13)	11 (13)	24 (15)	4 (8)	0.48
**Chronic pulmonary disease^f^**	45 (15)	17 (20)	20 (13)	8 (17)	0.28
**Cerebrovascular disease^g^**	23 (8)	6 (7)	13 (8)	4 (8)	0.95
**Chronic liver disease^h^**	3 (1)	0	3 (2)	0	0.28
**Chronic renal failure^i^**	39 (13)	11 (13)	21 (13)	7 (15)	0.97
**Type of initial parenteral AC**					0.78
**Unfractionated heparin**	104 (36)	27 (32)	59 (37)	18 (38)	
**Low-molecular-weight heparin**	152 (52)	47 (56)	83 (52)	22 (46)	
**Fondaparinux**	27 (9)	8 (10)	12 (8)	7 (15)	
**No parenteral AC**	8 (3)	2 (2)	5 (3)	1 (2)	
**Duration of AC (months)**	17.1 (6.3; 34.4)	8.3 (5.6; 29.0)	20.1 (6.8; 35.3)	26.9 (8.1; 37.7)	0.003
**Use of inferior vena cava filter**	7 (2)	3 (4)	2 (1)	2 (4)	0.37
**Thrombolysis**	19 (7)	1 (1)	9 (6)	9 (19)	<0.001
**Massive PE^j^**	6 (2)	1 (1)	3 (2)	2 (4)	0.50
**PESI (points)**	97 (83; 115)	96 (83; 116)	97 (84; 116)	88 (77; 110)	0.08
**RV/LV diameter ratio**	1.1 (0.9; 1.3)	1.0 (0.9; 1.1)	1.1 (0.9; 1.3)	1.3 (1.1; 1.6)	<0.001

Abbreviations: CTOI, computed tomography obstruction index; PE, pulmonary embolism; DVT, deep vein thrombosis; BMI, body mass index; VTE, venous thromboembolism; AC, anticoagulation; PESI, Pulmonary Embolism Severity Index; RV, right ventricular; LV, left ventricular.

^a^At the time of the index VTE.

^b^Unprovoked PE was defined as PE in the absence of immobilization, major surgery, oral oestrogen therapy, or active cancer during the last three months before index PE.

^c^Major surgery, oestrogen therapy, immobilization (fracture or cast of the lower extremity, bed rest >72 hours, or voyage in sitting position for >6 hours) during the last 3 months before index PE.

^d^Cancer requiring surgery, chemotherapy, radiotherapy, or palliative care during the last 3 months before index PE.

^e^Systolic or diastolic heart failure, left or right heart failure, forward or backward heart failure, or a known left ventricular ejection fraction of <40%.

^f^Chronic obstructive pulmonary disease, active asthma, lung fibrosis, cystic fibrosis, or bronchiectasis.

^g^History of ischemic or haemorrhagic stroke with hemiparesis, hemiplegia, or paraplegia at the time of screening.

^h^Liver cirrhosis, chronic hepatitis, chronic liver failure or hemochromatosis. Fatty liver was not considered a chronic liver disease.

^i^Chronic renal failure requiring or not haemodialysis such as diabetic or hypertensive nephropathy, chronic glomerulonephritis, chronic interstitial nephritis, myeloma-related nephropathy, or cystic kidney disease.

^j^Defined as systolic blood pressure of <90 mm Hg at the time of PE diagnosis.

**Fig 1:**
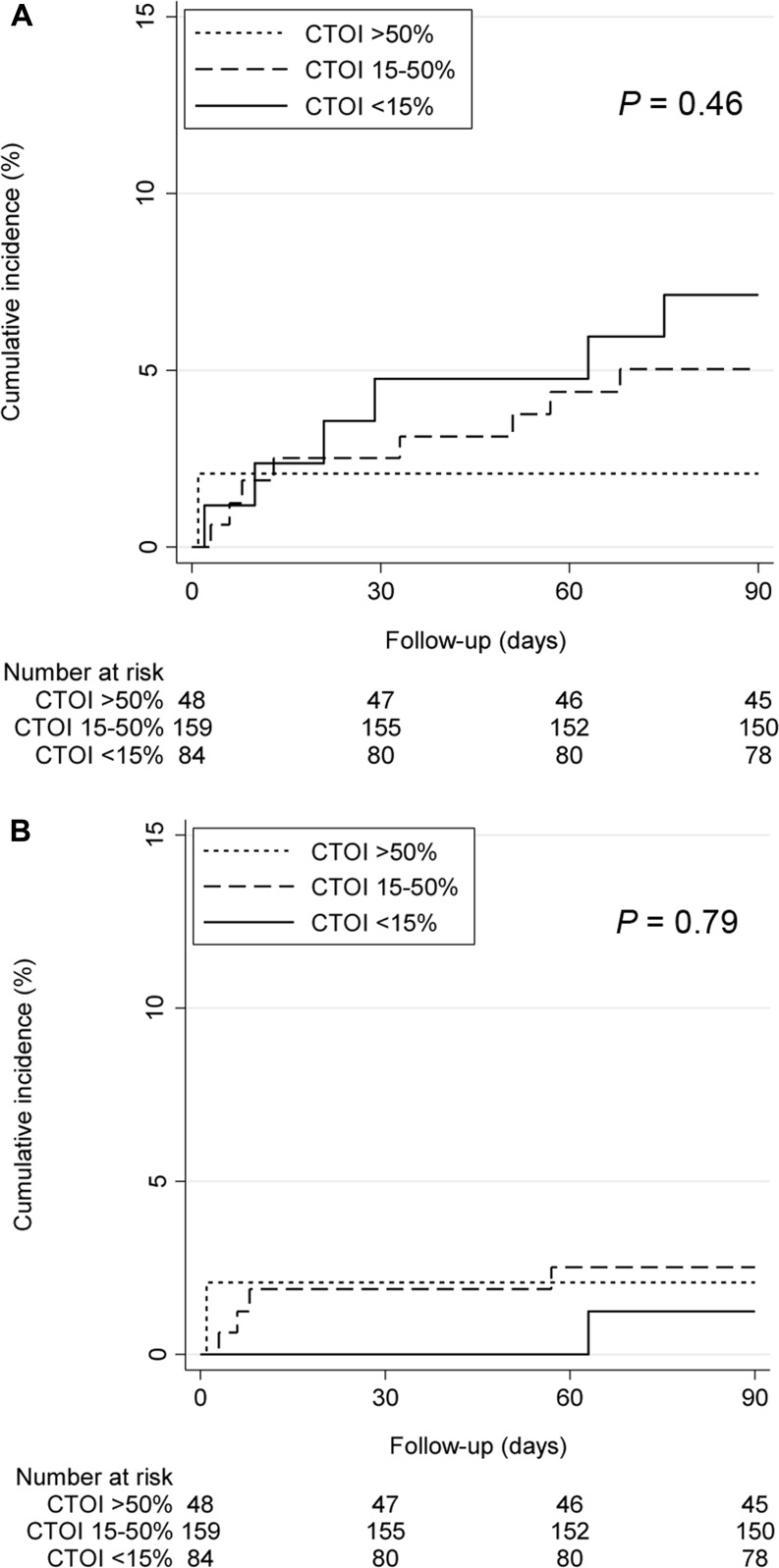
90-day mortality by CTOI (<15% versus 15–50% versus >50%). A. All deaths. B. PE-related deaths.

In the Kaplan-Meier analysis, the cumulative incidence of overall as well as PE-related death did not differ significantly between the different CTOI strata after 90 days (*P* = 0.46 and *P* = 0.79, respectively) (Fig 1). However, there was a significant association between continuous CTOI and PE-related 90-day mortality (adjusted sub-hazard ratio [SHR] per 10% CTOI increase 1.36; 95% confidence interval [CI] 1.03–1.81; *P* = 0.03), but not between continuous CTOI and 90-day overall mortality (adjusted hazard ratio [HR] per 10% CTOI increase 0.92; 95% CI 0.70–1.21; *P* = 0.54) (Table 2). In contrast to the PESI, the RV/LV diameter ratio was not associated with overall and PE-related 90-day mortality (Table 2).

**Table 2:** Association of CTOI, RV/LV diameter ratio, and PESI with 90-day mortality.

**Overall mortality**	**Crude HR or SHR (95% CI)**	***P* value**	**Adjusted HR or SHR (95% CI)**	***P* value**
CTOI (per 10%)	0.90 (0.70; 1.14)	0.37	0.92 (0.70; 1.21)^a^	0.54
RV/LV diameter ratio (per unit)	0.27 (0.03; 2.16)	0.22	0.35 (0.06; 2.18)^a^	0.26
PESI (per 10 points)	1.36 (1.21; 1.53)	<0.001	1.34 (1.19; 1.51)^b^	<0.001
**PE-related mortality**				
CTOI (per 10%)	1.33 (1.00; 1.78)	0.05	1.36 (1.03; 1.81)^a^	0.03
RV/LV diameter ratio (per unit)	0.61 (0.17; 2.25)	0.46	0.69 (0.23; 2.07)^a^	0.51
PESI (per 10 points)	1.15 (1.04; 1.28)	0.01	1.12 (1.01; 1.26)^b^	0.04

Abbreviations: CTOI, computed tomography obstruction index; RV, right ventricular; LV, left ventricular; PESI, Pulmonary Embolism Severity Index; PE, pulmonary embolism; HR, hazard ratio; SHR, sub-hazard ratio; CI, confidence interval.

^a^Adjustment was done for provoked PE, the PESI, and anticoagulation treatment as a time-varying covariate.

^b^Adjustment was done for provoked PE, and anticoagulation treatment as a time-varying covariate.

The CTOI had a poor, the PESI a good predictive accuracy for 90-day overall mortality (C-statistics 0.43 and 0.79, 95% CI 0.28–0.57 and 0.71–0.87, respectively). Further, the CTOI and the PESI had a similar predictive accuracy for PE-related 90-day mortality (C-statistics 0.69 and 0.70, 95% CI 0.52–0.85 and 0.61–0.79, respectively). In contrast, the predictive accuracy of the RV/LV diameter ratio for overall and PE-related 90-day mortality was poor (C-statistics 0.39 and 0.51, 95% CI 0.25–0.52 and 0.36–0.66, respectively).

**Fig 2:**
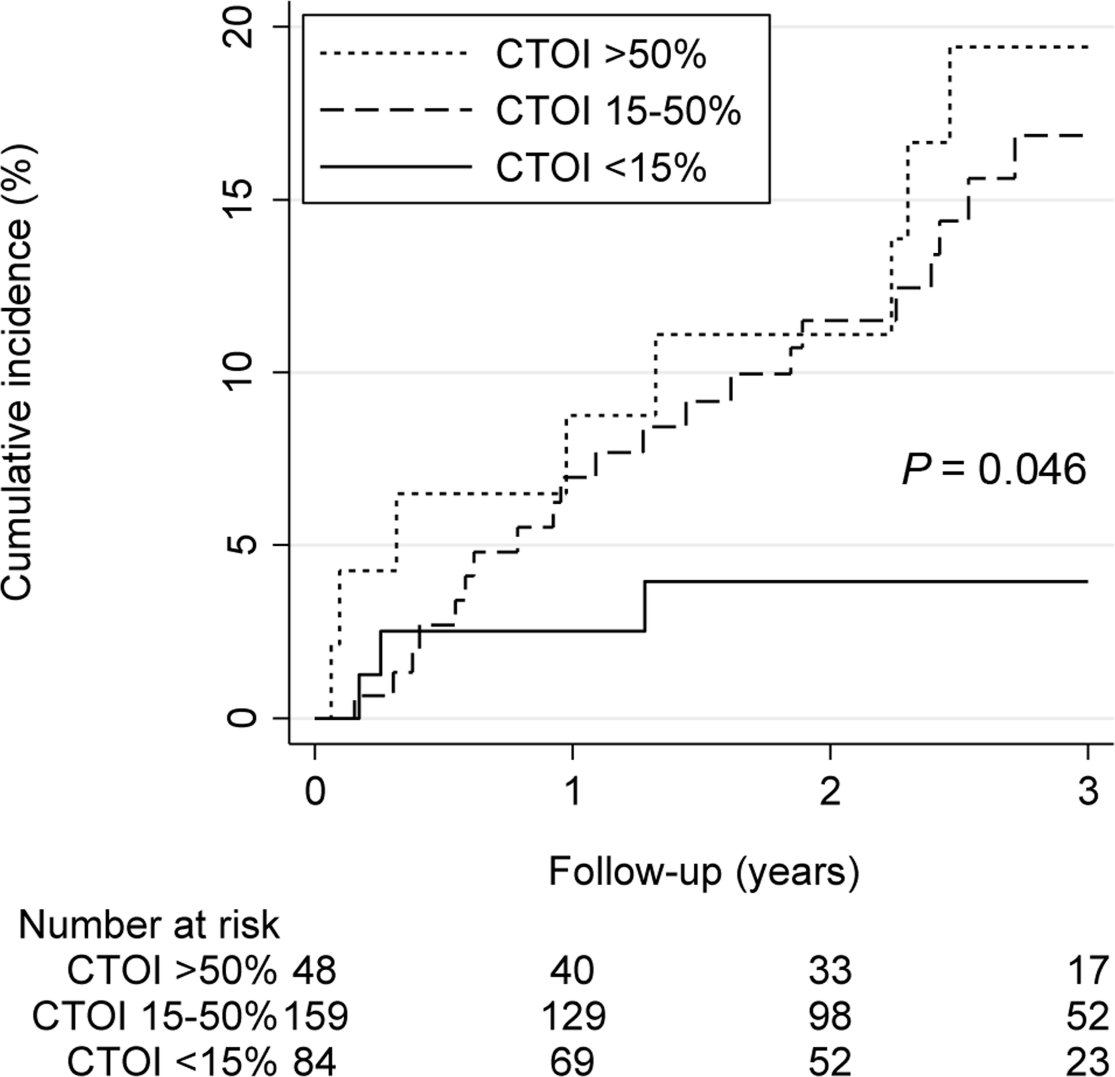
VTE recurrence by CTOI (<15% versus 15–50% versus >50%).

The cumulative incidence of VTE recurrence after 3 years differed significantly between different CTOI strata (*P* = 0.046) (Fig 2). Further, there was a significant association between continuous CTOI and VTE recurrence during the whole follow-up (adjusted SHR per 10% CTOI increase 1.27; 95% CI 1.12–1.45; *P* < 0.001), as well as between the RV/LV diameter ratio and VTE recurrence (adjusted SHR per unit increase 2.74; 95% CI 1.26–5.95; *P* = 0.01) (Table 3). The discriminative power of the CTOI (C-statistics 0.63; 95%CI 0.55–0.72) and the RV/LV diameter ratio (C-statistics 0.59; 95% CI 0.49–0.68) for VTE recurrence was moderate.

**Table 3:** Association of CTOI, RV/LV diameter ratio, and PESI with secondary outcomes.

**VTE recurrence**	**Crude SHR or time ratio (95% CI)**	***P* value**	**Adjusted SHR or time ratio (95% CI)**	***P* value**
CTOI (per 10%)	1.21 (1.07; 1.38)	0.003	1.27 (1.12; 1.45)^a^	<0.001
RV/LV diameter ratio (per unit)	1.56 (0.67; 3.67)	0.31	2.74 (1.26; 5.95)^a^	0.01
PESI (per 10 points)	1.02 (0.92; 1.14)	0.67	1.09 (0.97; 1.23)^b^	0.17
**Length of stay**				
CTOI (per 10%)	1.05 (1.01; 1.10)	0.02	1.06 (1.02; 1.11)^c^	0.01
RV/LV diameter ratio (per unit)	1.42 (1.11; 1.83)	0.01	1.36 (1.08; 1.72)^c^	0.01
PESI (per 10 points)	1.10 (1.07; 1.14)	<0.001	1.11 (1.07; 1.16)^d^	<0.001

Abbreviations: CTOI, computed tomography obstruction index; RV, right ventricular; LV, left ventricular; PESI, Pulmonary Embolism Severity Index; PE, pulmonary embolism; SHR, sub-hazard ratio; CI, confidence interval.

^a^Adjustment was done for provoked PE, the PESI, and anticoagulation treatment as a time-varying covariate.

^b^Adjustment was done for provoked PE, and anticoagulation treatment as a time-varying covariate.

^c^Adjustment was done for age, gender, type of PE (unprovoked versus provoked versus cancer-related), BMI, prior VTE, central PE, concomitant DVT, hypertension, diabetes, heart failure, chronic lung disease, cerebrovascular disease, chronic liver disease, chronic renal disease, the PESI, and antiplatelet therapy/antiplatelet therapy/non-steroidal anti-inflammatory drugs.

^d^Adjustment was done for age, gender, type of PE (unprovoked versus provoked versus cancer-related), BMI, prior VTE, central PE, concomitant DVT, hypertension, diabetes, heart failure, chronic lung disease, cerebrovascular disease, chronic liver disease, chronic renal disease, and antiplatelet therapy/antiplatelet therapy/non-steroidal anti-inflammatory drugs.

The CTOI (adjusted time ratio (TR) per 10% CTOI increase 1.06; 95% CI 1.02–1.11; *P* = 0.01), the RV/LV diameter ratio (adjusted TR per unit increase 1.36; 95% CI 1.08–1.72; *P* = 0.01) and the PESI (adjusted TR per 10 points increase 1.11; 95% CI 1.07–1.16; *P* < 0.001) were significantly associated with LOS for patients admitted to hospital due to index PE.

## Discussion

In our prospective cohort of elderly patients with acute symptomatic PE, the CTOI was not associated with all-cause mortality, but with PE-related mortality at 90 days. Neither all-cause nor PE-related mortality differed significantly across the three pre-specified CTOI strata. The discriminative power of the CTOI for predicting PE-related 90-day mortality was moderate (C-statistics 0.69). In contrast, the RV/LV diameter ratio was neither associated with overall nor PE-related 90-day mortality.

The predictive value of the CTOI for short-term overall mortality was previously assessed only in few prospective studies and one meta-analysis, which all showed no association between the CTOI and 30-day mortality or clinical deterioration (cardiopulmonary resuscitation, mechanical ventilation, administration of inotropic or thrombolytic agents) in the hospital [6, 14–16]. None of these studies assessed PE-related death. While our results did not show an association between CTOI and overall mortality, the CTOI was significantly associated with PE-related mortality. This is not astonishing. Given the fact that elderly patients with VTE are often multimorbid, other clinical factors may be more influential with respect to survival than the embolic burden alone [29, 30]. Despite its association with PE-related mortality, the CTOI does not appear to offer any advantage over the PESI in terms of mortality prediction.

In contrast to the results of three meta-analyses [7–9], RV/LV diameter ratio assessed by CTPA was not associated with mortality in our prospective cohort. Overall, only 8 of 39 studies included in these meta-analyses were prospective and according to a meta-regression analysis, the risk estimates derived from retrospective studies were significantly higher than the estimates from the prospective studies [9]. Moreover, patients in all prospective studies that demonstrated an association between RV/LV diameter ratio and mortality were younger than the patients enrolled in our cohort (mean age 54–67 versus 75.4 years) [17, 31, 32]. Because co-morbid conditions as well as ageing itself may lead to left ventricular enlargement or right ventricle dilatation, the prognostic accuracy of RV/LV diameter ratio may be lower in elderly multimorbid patients. Indeed, the largest prospective cohort including 848 mostly elderly patients (median age 72 years) did not demonstrate an association between RV/LV diameter ratio and mortality [18].

In contrast to two previous studies [33, 34], our study is the first to show an association between the CTOI and VTE recurrence. Den Exter et al. focused on the association between thromboembolic resolution assessed by CTPA and VTE recurrence, mentioning that the CTOI was not associated with recurrent VTE (no data shown) [33]. Zhang et al. showed no association between the CTOI and recurrent VTE in patients with mainly provoked VTE (67% versus 39% in our study), which may be a reason for the different findings. Further, their analysis on the association between the CTOI and VTE recurrence was lacking adjustment for time periods in which patients were anticoagulated during follow-up. Only few studies showed an association between echocardiography-assessed right ventricular dysfunction and VTE recurrence [35, 36]. To the best of our knowledge, our study is the first to confirm the relationship between CTPA-assessed right ventricular dysfunction and recurrent VTE. However, the clinical value of risk stratification for recurrent VTE by CTPA if at all would be limited to patients in whom optimal duration of anticoagulation is unclear (e.g., patients with unprovoked VTE).

All three measures of disease severity, the CTOI, RV/LV diameter ratio, and the PESI were associated with LOS in our study. Given that treating physicians were not blinded to these measures in our study, the greater true or perceived severity of illness may have led to an extended hospital stay.

The strengths of our study include the prospective multicenter design, inclusion of consecutive patients with objectively diagnosed PE, blinded assessment of the CTOI, the outcome assessment by a blinded independent committee using pre-defined criteria, and the focus on elderly patients who are at particular risk of PE-related complications.

Our study has potential limitations. First, due to the low number of deaths (9 and 15 patients after 30 and 90 days, respectively) only a minimal adjustment and no analysis of the association between the CTOI and 30-day mortality could be performed. Second, our study might be underpowered to detect an association between the CTOI or RV/LV diameter ratio and all-cause mortality. Third, we enrolled exclusively patients aged 65 years or older with acute VTE. We thus cannot extrapolate our results to younger patients. Fourth, the fact that patients with a higher CTOI were more likely to receive thrombolytic therapy might have biased the results. However, when we excluded thrombolyzed patients in a sensitivity analysis, our results remained unchanged. Finally, we could not analyze the interobserver agreement for the CTOI assessment, because disagreement was immediately resolved by consensus and only the final CTOI value was available in our database.

In conclusion, our results showed that the CTOI is not associated with overall 90-day mortality in elderly patients with acute PE. However, we showed an association between the CTOI and PE-related 90-day mortality and VTE recurrence. The RV/LV diameter ratio was associated with recurrent VTE but not with overall or PE-related mortality.
